# Anti-IL23/12 agents and JAK inhibitors for inflammatory bowel disease

**DOI:** 10.3389/fimmu.2024.1393463

**Published:** 2024-07-17

**Authors:** Zhezhe Tian, Qiaorui Zhao, Xiu Teng

**Affiliations:** ^1^ Laboratory of Human Disease and Immunotherapies, West China Hospital, Sichuan University, Chengdu, China; ^2^ Hepatic Department of Hepatology, Qilu Hospital of Shandong University, Jinan, China; ^3^ State Key Laboratory of Biotherapy, West China Hospital, Sichuan University, Chengdu, China; ^4^ Institute of Immunology and Inflammation, Frontiers Science Center for Disease−Related Molecular Network, West China Hospital, Sichuan University, Chengdu, China

**Keywords:** inflammatory bowel disease, IL-12/23 p40 subunit, IL-23 p19 subunit, JAK/STAT signaling, anti-IL12/23 biologics, JAK inhibitors, proteolysis targeting chimera (PROTAC)

## Abstract

IBD (inflammatory bowel disease) is a chronic inflammatory disease of the gastrointestinal tract with increasing incidence worldwide. Multiple factors, such as genetic background, environmental and luminal factors, and mucosal immune dysregulation, have been implicated in the cause of IBD, although the cause of the disease remains unknown. IL-12 and IL-23 and their downstream signaling pathways participate in the pathogenesis of inflammatory bowel disease. Early and aggressive treatment with biologic therapies or novel small molecules is needed to decrease complications and the need for hospitalization and surgery. The landscape of inflammatory bowel disease (IBD) treatment has tremendously improved with the development of biologics and small molecule drugs. Several novel biologics and small molecule drugs targeting IL-12 and IL-23 and their downstream targets have shown positive efficacy and safety data in clinical trials, and several drugs have been approved for the treatment of IBD. In the future, numerous potential emerging therapeutic options for IBD treatment are believed to come to the fore, achieving disease cure.

## 1 Introduction

Inflammatory bowel disease (IBD) is a chronic intestinal disorder that encompasses two distinct disorders, Crohn’s disease (CD) and ulcerative colitis (UC) (1). Since the turn of the twenty-first century, the global prevalence of inflammatory bowel disease (IBD) has been increasing worldwide, with 1 in 200 individuals affected in Western countries (2). In China, IBD has gone from being a rare condition to one that is common and accounts for substantial use of hospital beds due to urbanization (2). The incidence and prevalence of both disorders are much lower in developing jurisdictions than in developed jurisdictions (3, 4). There are several reasons for this, including environmental factors, genetic factors and diagnosis bias. IBD is most commonly diagnosed in patients younger than 30 years, although the incidence of IBD in older individuals is increasing (2, 5, 6). Moreover, CD is predominantly observed in males in childhood but becomes predominant in women in adulthood, while the incidence of UC is reported to be equal between the sexes from childhood to adulthood (3).

Ulcerative colitis is restricted to the colon, and inflammation in UC is typically limited to the mucosal layer, causing superficial damage to the bowel wall (3). In contrast, Crohn’s disease is characterized by the involvement of the gastrointestinal tract from the mouth to the anus in a discontinuous fashion and transmural inflammation (involving all layers of the bowel wall) that leads to fibrosis, stricture and fistula (3, 7). Bloody diarrhea is the most common symptom of UC, whereas rectal bleeding or bloody diarrhea occurs in CD patients with colonic involvement (3, 4). The exact pathophysiology of IBD is still unclear, but several factors, including a dysregulated immune response, altered gut microbiota, genetic susceptibility and environmental factors, contribute to the risk of disease onset and progression (3, 8). In IBD, activated macrophages produce increased levels of interleukin-12 and interleukin-23, and dendritic cells present antigens to naive T cells, facilitating the differentiation of T helper 1 (Th1) cells and T helper 17(Th17)cells, which produce proinflammatory cytokines, such as IFN-γ and tumor necrosis factor (TNF) (1). Additionally, these proinflammatory cytokines are produced by ILC1s (innate lymphoid cells) and ILC3s upon activation by IL-12 and IL-23, promoting inflammation (9). JAK/STAT family members, as signaling pathways downstream of mediators such as IL-12 and IL-23, also play key roles in the pathophysiology of IBD (10). STAT3 and STAT4 have been shown to be essential for the differentiation of Th17 cells and Th1 cells, depending on STAT3 and STAT4 phosphorylation mediated by JAK2 and TYK2 in response to IL-12 and IL-23 (10).

A therapeutic strategy focused on precise molecular targeting of inflammatory cascades has improved the understanding of the complex pathophysiology of CD and UC (7). The therapeutic options have expanded substantially over the past decade with the development of biologics, including antibodies for the inhibition of IL-23 and IL-12 and small molecules such as JAK inhibitors (**Table 1**), allowing more stringent therapeutic goals to be considered, including clinical and endoscopic remission (6). This review provides a comprehensive overview of the current knowledge of the pathophysiology and up-to-date therapeutic options targeting pathogenic cascades of the cytokine IL-12/23 and the downstream JAK/STAT pathway for IBD treatment.

**Table 1:** JAK inhibitors for IBD treatment.

Drug	Status	Clinical trials	Clinical use	Inhibits	Company
Tofacitinib	approved	NCT03281304(TERMINATED) NCT01465763(COMPLETED) NCT01458951(COMPLETED) NCT03885713 (RECRUITING) NCT04424303 (RECRUITING) NCT04624230 (RECRUITING) NCT04925973 (ACTIVE, NOT RECRUITING) NCT05313620 (RECRUITING) NCT04338204 (ACTIVE, NOT RECRUITING) NCT05112263 (NOT YET RECRUITING) NCT05728008 (COMPLETED) NCT05431283 (RECRUITING) NCT03103412 (COMPLETED) NCT04505410 (RECRUITING) NCT01393899 (COMPLETED) NCT01393626 (COMPLETED) NCT01458574 (COMPLETED) NCT01470599 (COMPLETED) NCT00615199 (COMPLETED) NCT01470612 (COMPLETED)	CD、UC	JAK1, JAK3, JAK2	Pfizer Inc.
TD-1473 izencitinib	phase3	NCT03920254 (TERMINATED) NCT03758443 (TERMINATED) NCT03635112 (TERMINATED) NCT03920254 (TERMINATED)	CD、UC	JAK1, JAK3, JAK2,TYK2	Janssen
Peficitinib	phase2	NCT01959282 (COMPLETED)	UC	JAK1, JAK3, JAK2,TYK2	Astellas Pharma Inc.
Upadacitinib	approved	NCT03345836 (COMPLETED) NCT03345849 (COMPLETED) NCT03006068 (ACTIVE, NOT RECRUITING) NCT05867329 (RECRUITING) NCT03345823 (ACTIVE, NOT RECRUITING) NCT05782907 (RECRUITING) NCT03653026 (COMPLETED) NCT02819635 (COMPLETED) NCT02782663 (ACTIVE, NOT RECRUITING) NCT02365649 (COMPLETED)	CD、UC	JAK1	Abbvie
Filgotinib	phase3	NCT03201445 (ACTIVE, NOT RECRUITING) NCT03046056 (COMPLETED) NCT02914600 (TERMINATED) NCT03077412 (COMPLETED) NCT02914561 (COMPLETED) NCT02048618 (COMPLETED) NCT05817942 (RECRUITING) NCT05479058 (TERMINATED) NCT02914535 (ACTIVE, NOT RECRUITING) NCT02914522 (COMPLETED) NCT05653791 (ACTIVE, NOT RECRUITING) NCT06089590 (NOT YET RECRUITING)	CD、UC	JAK1	Gilead
SHR0302	phase3	NCT03677648 (COMPLETED) NCT05181137 (RECRUITING) NCT03675477 (COMPLETED)	CD、UC	JAK1	Jiangsu Hengrui Pharmaceuticals Co.,Ltd.
Itacitinib	phase2	NCT03627052 (WITHDRAWN)	UC	JAK1	Incyte Corp.
Ritlecitinib (PF-06651600)	phase2	NCT02958865 (COMPLETED) NCT03395184 (COMPLETED) NCT02684760 (COMPLETED) NCT02309827 (COMPLETED)	CD、UC	JAK3/TEC	Pfizer Inc.
PF-06700841	phase2	NCT03395184 (COMPLETED) NCT02958865 (COMPLETED)	CD、UC	TYK2, JAK1	Pfizer Inc.
Deucravacitinib (BMS-986165)	phase2	NCT03262727 (COMPLETED) NCT03254784 (COMPLETED) NCT03599622 (ACTIVE, NOT RECRUITING) NCT04613518 (ACTIVE, NOT RECRUITING) NCT03934216 (COMPLETED) NCT04877990 (COMPLETED)	CD、UC	TYK2	BMS

## 2 Pathophysiology

**Figure 1:**
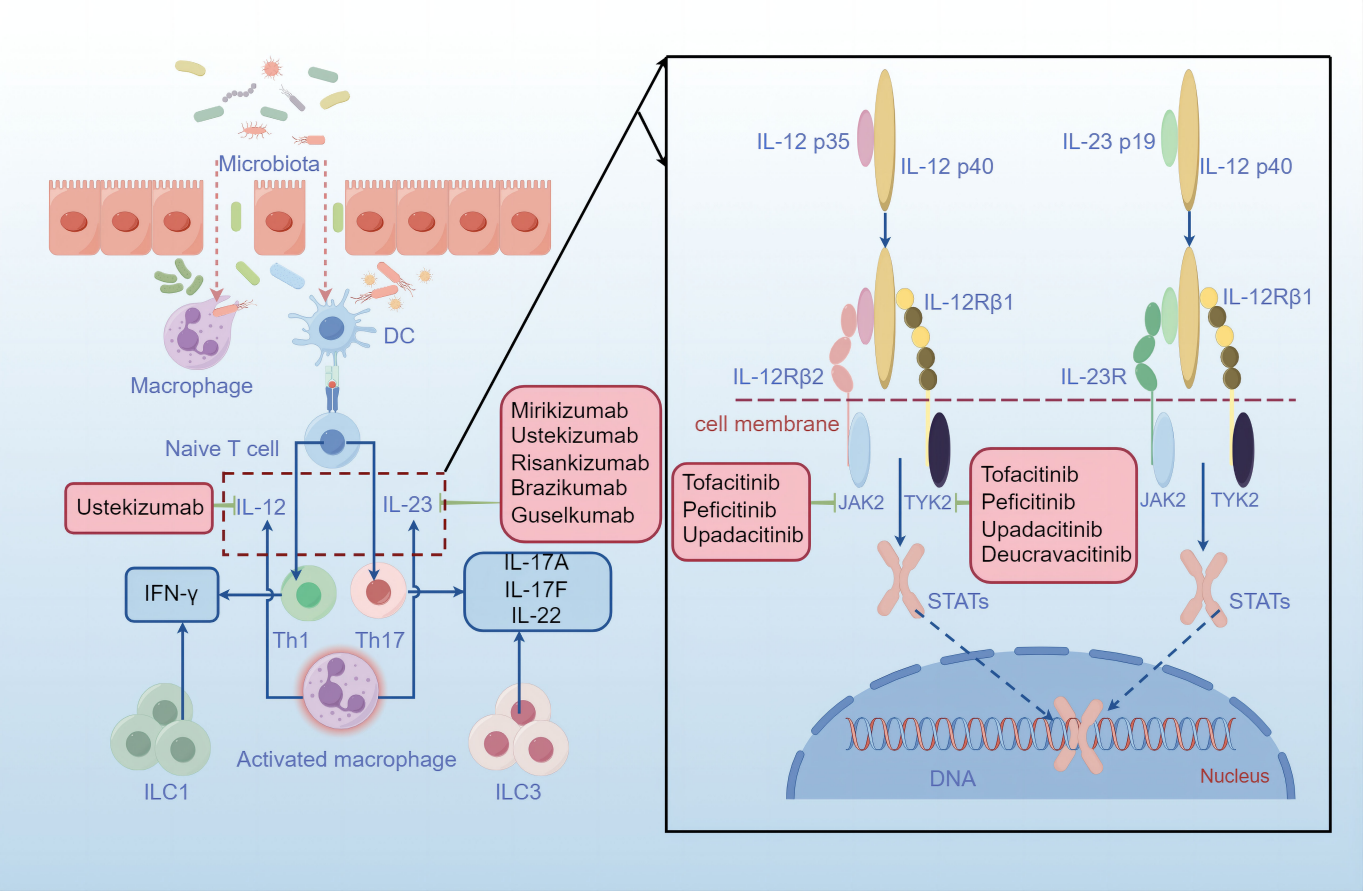
Mucosal injury and bowel inflammation. The intestinal epithelium is disrupted in IBD, thereby permitting more microbiota to cross the barrier, activating macrophages and antigen-presenting cells (APCs). Activated macrophages engulf the microbiota and produce increased levels of interleukin-23 and interleukin-12, resulting in the polarization of Th1 cells and Th17 cells, which produce proinflammatory cytokines such as IFN-γ, IL-17A, IL-17F and IL-22. After contact with an antigen, antigen-presenting cells (APCs), such as dendritic cells, present antigen to naive T cells, which proliferate and differentiate into effector T-cell subsets, such as Th1 cells and Th17 cells. Additionally, ILCs contribute to cytokine production and inflammation. IL-12 and IL-23 are heterodimeric cytokines that share an IL-12p40 subunit. IL-12p35 binds to its receptor IL-12Rβ2, and IL-23p19 binds to IL-23R, leading to structural alterations that facilitate high-affinity association of the IL-12p40 subunit with the IL-12Rβ1 chain. Thus, JAK2 and TYK2 are activated, triggering the phosphorylation and homodimerization of STATs, which translocate into the nucleus and activate distinct transcriptional programs. Treatment strategies in various phases of clinical development are shown in pink boxes. The monoclonal antibody ustekinumab can bind to the IL-12p40 subunit, interfering with both IL-12 and IL-23 signaling. In addition, several antibodies, including risankizumab, brazikumab, mirikizumab and guselkumab, recognize the IL-23p19 subunit and are being evaluated in clinical trials in patients with Crohn’s disease or ulcerative colitis. Moreover, JAK inhibitors such as tofacitinib, peficitinib, upadacitinib and decucravacitinib are also good options for IBD treatment.

The pathophysiology of IBD is multifaceted and incompletely understood. However, the prevailing view is that the pathogenesis of IBD is caused by genetic, immunological and environmental factors, leading to an abnormal immune system response to the intestinal microbiome (11) (**Figure 1**). More than 240 genetic loci involved in pathways recognizing microbial products, the autophagy pathway, the regulation of epithelial barrier function and pathways controlling innate and adaptive immunity have been associated with IBD (1, 12). During mucosal injury and inflammation in IBD, the epithelial barrier is breached as a primary or secondary event, and increased bacterial exposure elicits a proinflammatory immune response via dendritic cells and inflammatory macrophages. An improved understanding of the mucosal immune system has led to an expanding array of therapeutic targets. As mentioned above, the IL-12/23 and JAK/STAT pathways are highly important for the immune response in IBD.

Interleukin-12 (IL-12) and interleukin-23 (IL-23), which are produced by antigen-presenting cells (APCs), such as dendritic cells and macrophages, play key roles in the pathogenesis of inflammatory bowel disease (9, 13). The process by which APCs produce IL-12 is also driven by the CD40 ligand on T cells, which is modulated by cytokines such as IFN-γ, IL-18, and IL-4 and by IL-12 itself. Additionally, CD40 stimulation can lead to the production of IL-23 by APCs (13). IL-12, a heterodimeric cytokine composed of interleukin-12p35 and interleukin-12p40 subunits, functions in the biological differentiation of naive CD4+ T cells into Th1 cells, which produce interferon-γ, resulting in inflammation (1, 9). Notably, the ILC subset is more abundant in the inflamed tissues of patients with Crohn’s disease than in those of healthy individuals, and dendritic cell-derived IL-12 stimulates group 1 ILCs to produce type 1 cytokines such as IFN-γ and TNF (9). Similarly, IL-23, a heterodimeric cytokine composed of interleukin-23p19 and interleukin-12p40 subunits, differentiates naive CD4+ T cells into Th17 cells, which recruit neutrophils and produce interleukin-17A, interleukin-17F, and interleukin-22 (9). IL-23 also stimulates other type 17 cells, such as ILC3s, which produce IL-17 family cytokines, whose expression is increased in ILCs isolated from the inflamed colon of patients with Crohn’s disease (9, 14).

The JAK family includes JAK1, JAK2, JAK3 and tyrosine kinase 2 (TYK2) (10). Members of the JAK/STAT family mediate signaling via IL-12R and IL-23R, and both receptors interact with JAK family members, mainly JAK2 and TYK2 (9). Binding of IL-12p35 to its receptor IL-12Rβ2 or of IL-23p19 to IL-23R results in structural alterations that facilitate high-affinity association of the IL-12p40 subunit with the IL-12Rβ1 chain, inducing the activation of JAK2 and TYK2 (9). The phosphorylation and homodimerization of STAT4 result from the activation of the IL-12 receptor complex composed of IL-12Rβ1 and IL-12Rβ2, while the formation of STAT3 and STAT4 homodimers is due to IL-23 receptor signaling; thus, STAT3 is translocated into the nucleus, where it performs its functions (9). In naive CD4+ T cells, STAT4 signaling together with the transcription factor T-bet induces differentiation toward the Th1 cell phenotype and the production of interferon-γ (13). However, upon IL-23 ligation, STAT3 signaling initiates and enhances the activity of the lineage-specific transcription factor retinoid-related orphan receptor-γt in Th17 cells.

Therefore, various antibodies blocking the activity of inflammatory cytokines and JAK inhibitors have shown positive results in clinical trials undertaken in patients with IBD, and these agents have led to a new era in IBD therapy (15, 16).

## 3 Anti-IL-23/12 agents

### 3.1 Mirikizumab

Mirikizumab is a humanized IgG4-variant monoclonal antibody against interleukin-23 p19 that can block the binding of IL-23 and its receptor to prevent the activation of cellular effector functions through the JAK/STAT pathway (17). It was the first IL-23p19 inhibitor approved for use in the treatment of moderate-to-severe UC patients who had an inadequate response or a lost response or who were intolerant to either conventional therapy (aminosalicylates, corticosteroids, immunomodulators, or tofacitinib) or a biologic treatment (anti-TNF therapy) (18, 19). Data from two randomized, placebo-controlled phase III trials of mirikizumab in adults with moderately to severely active ulcerative colitis showed that patients met the primary endpoint of clinical remission at week 12 in the induction trial (*P* < 0.001, 24.2% versus 13.3% of patients in the mirikizumab group and the placebo group) and week 40 in the following maintenance program (49.9% versus 25.1%, *P* < 0.001) (20, 21). Additionally, compared with placebo, mirikizumab led to histologic-endoscopic remission in moderate-to-severe UC patients and achieved bowel urgency relief (22, 23). Additionally, in an earlier phase II study of patients with UC, mirikizumab demonstrated greater efficacy and durable safety throughout the induction and maintenance period (clinicaltrials.gov no: NCT02589665) (24). Despite focusing on the indications for UC and CD, mirikizumab achieved efficacy endpoints in patients with psoriasis in the phase III OASIS program (NCT03482011, NCT03535194 and NCT03556202) (18, 25). Additionally, some mild adverse reactions and nonmelanoma skin cancers can occur in mirikizumab recipients (18).

### 3.2 Ustekinumab

Ustekinumab, a humanized immunoglobulin (Ig)G1κ monoclonal antibody directed against the shared p40 subunit of interleukins 12 and 23, has been approved for the treatment of CD, UC, psoriatic arthritis (PsA) and moderate to severe plaque psoriasis (26, 27). The IM-UNITI study and long-term extension (LTE) (ClinicalTrials.gov no: NCT01369355) showed that long-term subcutaneous ustekinumab therapy was well tolerated and effective at maintaining clinical remission through 5 years in patients who were TNF antagonist-naive, as well as those who did not respond to, had lost response to, or were intolerant of TNF antagonists (28). In that trial, 84.8% and 90.0% of the patients who were treated with ustekinumab every 12 weeks or 8 weeks, respectively, were in clinical remission at week 252 with the absence of new safety signals (28). The efficacy and safety of ustekinumab in patients with ulcerative colitis were confirmed in another three-year program called the UNIFI long-term extension (LTE) study (ClinicalTrials.gov no: NCT02407236) (29). Active-comparator trials or indirect evaluation studies have suggested that compared with adalimumab, infliximab and risankizumab, ustekinumab treatment is more effective for patients with moderately to severely active CD (30–32). In addition to treating IBD, ustekinumab has shown long-term effectiveness in treating psoriatic arthritis (33) and has been approved for treating moderate to severe plaque psoriasis (34). Nevertheless, this drug also has several side effects, such as anal abscess, pneumonia, cellulitis, diverticulitis, gastroenteritis, abdominal abscess, perirectal abscess, pyelonephritis, sepsis, and cholecystitis (28). Because of the lack of long-term studies on the use of mirikizumab, ustekinumab could be a better choice than mirikizumab for the treatment of IBD.

### 3.3 Risankizumab

Risankizumab is a humanized IgG1 monoclonal antibody that can specifically bind to the p19 subunit of IL-23 to prevent it from interacting with the IL-23R complex (35) and has been approved for the treatment of Crohn’s disease (36). In phase 3 induction trials called ADVANCE (ClinicalTrials.gov no: NCT03105128) and MOTIVATE (ClinicalTrials.gov no: NCT03104413), a greater proportion of moderate-to-severe CD patients with and without previous bio-failure reached the coprimary endpoints, including clinical remission and endoscopic response, for risankizumab than for placebo (35). Similarly, a maintenance trial named FORTIF (ClinicalTrials.gov no: NCT03105102), in which patients from the two above trials were included, indicated that risankizumab had a certain degree of durability according to the increased proportion of patients in clinical remission and endoscopic response until week 52 (37).

### 3.4 Guselkumab

Guselkumab, an interleukin-23p19 subunit antagonist, is approved for the treatment of plaque psoriasis and psoriatic arthritis (38). Two phase 2 trials indicated that guselkumab induced greater clinical and endoscopic improvements in patients with CD or UC with inadequate response or intolerance to conventional or biologic therapy, with a favorable safety profile (38, 39). Moreover, phase 3 studies evaluating the efficacy and safety of guselkumab for the treatment of patients with IBD are currently in progress (38) (clinicaltrials.gov).

### 3.5 Brazikumab

Brazikumab is also a human immunoglobulin G2 monoclonal antibody that binds to the p19 subunit of IL-23 to block the binding of IL-23 to its receptor (40). A phase 2a study provided evidence for the significant clinical and biological effects of brazikumab as an induction therapy for CD refractory to TNF antagonists (40). However, the studies of brazikumab in IBD were terminated for business reasons (NCT02574637 and NCT03616821).

## 4 JAK inhibitors

### 4.1 Tofacitinib

Tofacitinib is an oral pan-Jak inhibitor that binds to JAK1, JAK2, and JAK3, thereby weakening the downstream effects of several IBD-associated cytokines (41). It was approved for the treatment of UC in 2018 on the basis of three randomized, double-blind, placebo-controlled trials (the OCTAVE 1 and 2 induction trials and the OCTAVE Sustain maintenance trial) (7, 10). According to these three trials (NCT01465763, NCT01458951, and NCT01458574), tofacitinib was more effective as an induction and maintenance therapy than was a placebo in patients with moderately to severely active ulcerative colitis (42). In addition, although primary efficacy endpoints were not significantly different from those of placebo in phase 2b trials (NCT01393626 and NCT01393899), a degree of clinical efficacy for tofacitinib was observed in inducing and maintaining remission in moderate-to-severe CD patients, supporting further investigation of the efficacy and safety of JAK inhibition for CD (43). Adverse events of tofacitinib included venous thromboembolism, herpes zoster, and major adverse cardiovascular events (11).

### 4.2 Peficitinib

Peficitinib is an oral JAK1, JAK2, JAK3 and Tyk2 (pan-JAK) inhibitor that has shown positive efficacy in IBD treatment. In a phase 2 trial (NCT01959282), a greater proportion of UC patients receiving peficitinib achieved clinical response, remission, and mucosal healing at week 8 than did those receiving placebo. Additionally, trends toward increased rates of primary endpoints were observed at week 8 in moderately-to-severely active UC patients treated with higher doses (44). However, higher and more frequent treatment-emergent adverse event rates were reported in the combined peficitinib group than in the placebo group (44). Therefore, additional clinical trials are needed to further clarify the efficacy and safety of peficitinib in treating UC. Additionally, peficitinib was approved for the treatment of rheumatoid arthritis (45).

### 4.3 Upadacitinib

Upadacitinib is an orally administered, selective, reversible inhibitor of JAK1 that has greater inhibitory effects on JAK1 than on JAK2, JAK3, or TYK2. It has been approved for the treatment of CD and UC (46–48). Upadacitinib is a treatment option for patients with moderately to severely active ulcerative colitis due to its positive efficacy and safety profile, as demonstrated by phase 3 clinical trials (NCT02819635 and NCT03653026) (49). For patients with moderate-to-severe CD, upadacitinib induction and maintenance treatment was superior to placebo in three phase III trials (NCT03345849, NCT03345836, and NCT03345823) (50). Moreover, a phase 4 trial (NCT05867329) is being conducted to test the efficacy of upadacitinib for the treatment of patients with acute severe UC. Adverse events, including serious infections, opportunistic infections, anemia, neutropenia, and creatine kinase elevation, were observed more frequently in patients who received upadacitinib than in those who received placebo (50).

### 4.4 Ritlecitinib (PF-06651600)

Ritlecitinib is an irreversible JAK3 and TEC kinase family (BTK, BMX, ITK, RLK, TEC) inhibitor that may become a better choice for IBD treatment (51). A phase 2b, randomized, umbrella trial (NCT02958865) of patients with moderate-to-severe active ulcerative colitis suggested that ritlecitinib induction therapy was more effective than placebo. At week 8, the rates of clinical remission, modified clinical remission, endoscopic improvement, histologic improvement, and mucosal healing were greater in patients who received ritlecitinib than in those who received placebo (52). In addition, ritlecitinib showed acceptable short-term safety profiles for the treatment of moderate-to-severe active ulcerative colitis (52).

### 4.5 Deucravacitinib (BMS-986165)

Deucravacitinib, also known as BMS-986165, is a first-in-class, highly selective oral tyrosine kinase 2 (TYK2) inhibitor that has shown efficacy in animal tests (53). However, deucravacitinib failed in a phase 2 trial (NCT03934216) for UC, and its efficacy for treating CD remains unclear (53). Therefore, more trials are needed to confirm the therapeutic efficacy of deucravacitinib for IBD. It was also approved for the treatment of adults with moderate-to-severe plaque psoriasis in 2022 (54).

## 5 STAT inhibitors

The influence of the STAT family of proteins on IBD pathology is also an area of active investigation. No clinical trials have been undertaken for the treatment of IBD to date, although the pharmacokinetics and safety/tolerability of inhibitors targeting dual phosphorylation or the SH2 domain of STAT3 have been evaluated in patients with solid tumors and natural killer/T-cell lymphoma (55–59) (ClinicalTrials.gov no: NCT03240939). As technology advances, such as for the degradation of STAT proteins, more drugs targeting STAT could enter clinical development, adding to the IBD treatment armamentarium (60).

## 6 Outlook

Inflammatory bowel disease (IBD), which is typically classified as either ulcerative colitis or Crohn’s disease, is a chronic, immune-mediated, inflammatory disease of the digestive tract. The field of IBD therapeutics has undergone tremendous improvements with the development of new drugs that target various pathways, such as the IL-12/IL-23 pathway and the JAK/STAT pathway. Most of these new drugs are in advanced phases of study and have shown promising efficacy in patients with IBD. Although JAK-targeting small molecules are in early phase trials, they represent novel therapeutic options for IBD treatment. The emergence of these novel drugs means that we have a large number of available options, and they might enable new strict endpoints in IBD therapy to be achieved. In addition, further studies comparing the clinical response rate of these approved agents with that of traditional agents are necessary to obtain accurate results, and long-term safety data are needed. With the introduction of additional approved drugs in the future, it will be crucial to identify biomarkers to predict and monitor clinical success to enable personalized therapy in IBD patients.

## Author contributions

ZT: Writing – original draft, Writing – review & editing. QZ: Writing – review & editing. XT: Writing – review & editing, Conceptualization, Funding acquisition, Investigation, Methodology, Project administration, Software, Supervision, Validation, Visualization.
